# New model predicts in-hospital complications in myocardial infarction

**DOI:** 10.15190/d.2022.1

**Published:** 2022-03-04

**Authors:** Geovedy Martinez-Garcia, Miguel Rodriguez-Ramos, Maikel Santos-Medina, Annia Maria Carrero-Vazquez, Yanitsy Chipi-Rodriguez

**Affiliations:** ^1^Cardiology Service, Enrique Cabrera General Teaching Hospital, Havana, Cuba; ^2^Cardiology Service, Camilo Cienfuegos General Teaching Hospital, Sancti Spiritus, Cuba; ^3^Cardiology Service, Dr. Ernesto Guevara de La Serna General Teaching Hospital, Las Tunas, Cuba

**Keywords:** ST elevation myocardial infarction, in-hospital complications, leukoglycemic index, prediction.

## Abstract

INTRODUCTION AND OBJECTIVES: Ischemic cardiopathy constitutes the leading cause of death worldwide. Our aim was to evaluate the prognostic capacity of the leukoglycemic index as well as to create a predictive model of in-hospital complications in patients with ST elevation myocardial infarction. MATERIALS AND METHODS: This was a multicentral and cohort study, which included patients inserted in the Cuban Registry of acute myocardial infarction. The study investigated 900 patients with a validation population represented by 233 external subjects. In order to define the performance of the leukoglycemic index were evaluated the discrimination with the statistical C and the calibration by Hosmer – Lemeshow test. A model of logistic binary regression was employed in order to define the predictive factors. RESULTS: Optimal cut point of the leukoglycemic index to predict in-hospital complications was 1188 (sensibility 60%; specificity 61.6%; area under the curve 0.623; p < 0.001). In-hospital complications were significantly higher in the group with the leukoglycemic index ≥ 1188; a higher value was significantly associated with a higher risk to develop an in-hospital complication [RR (IC 95%) = 2.4 (1.804–3.080); p<0.001]. The predictive model proposed is composed by the following factors: age ≥ 66 years, leukoglycemic index ≥ 1188, Killip-Kimball classification ≥ II and medical history of hypertension. This scale had a good discrimination in both, the training and the validation population. CONCLUSION: The leukoglycemic index possesses a low performance when used to assess the risks for in hospital complications in patients with ST elevation myocardial infarction. The new predictive model has a good performance, which can be applied to estimate risk of in-hospital complications. This model would be able to contribute to the health systems of developing countries without additional cost; it enables prediction of the patients having a higher risk of complications and a negative outcome during the hospitable admission.

## INTRODUCTION

Ischemic cardiopathy constitutes the leading cause of death in the world. According to the World Health Organization, from the year 2000 the deceases for this disease have progressively increased. In 2016, the disease claimed over nine million lives^1^. Approximately seven million people die every year as a consequence of this disease in worldwide, that corresponds to 12.8% of all deaths^2^. In Cuba, heart ischemic diseases reached a death rate in the year 2020 of 165.8 deceases for each 100 000 inhabitants. Within them, the myocardial infarction occupies a preponderant place, with a dead rate of 69.7 for 100 000 inhabitants, higher than the numbers of the preceding year^3^.The incidence of in-hospital complications has diminished strongly after ST segment elevation myocardial infarction (STEMI) because of percutaneous coronary intervention (PCI) and medical treatment. However, this appears to obscure the patient’s prognosis.Recently, research on the use of new cardiovascular markers has increased, with the objective to help to identify and stratify patients with greater risk and worse prognosis after suffering STEMI. However, inaccessibility to most of these markers in Cuba, limit its usefulness in habitual clinical practice. In several studies, it has been shown that the leukocyte and blood glucose count are related to the genesis and progression of atherosclerotic disease as well as with the extension and complications of STEMI^4-9^.Quiroga and al. were the first investigators in propose the leukoglycemic index (LGI) as a prognostic marker of death and in-hospital complications in patients with STEMI^10^. We hypothesized that the LGI, measured in the first 24 hours of hospital admission, is a valid predictor of in-hospital complications in patients with STEMI. The purpose of the study was to evaluate the LGI’s predictive capability and to create a predictive model.

## MATERIALS AND METHODS

### Type of study and participants

An analytical, observational, multicenter, longitudinal and prospective cohort study was carried out, with data stored in the Cuban Registry of Acute Myocardial Infarction (RECUIMA)^11,12^. In this cohort 900 patients diagnosed with STEMI from January 2018 to the January 2020 were included with a consecutive form. The criteria of inclusion were: 1) diagnosis of STEMI, according to the 4th Universal Definition of Myocardial Infarction^13^; 2) venous blood samples were obtained in the first 24 hours after admitted. The sample was used to estimate white blood count (WBC) and fast plasma glucose (FPG); and 3) absence of inflammatory systemic, infectious or hematological known disease.As part of validation population 142 patients were recruited from January to July 2021. These patients satisfied the same criteria of inclusion previously exposed. Several clinical and epidemiological variables were extracted from the database: age, sex, medical history (diabetes mellitus, hypertension, dyslipidemia, smoking, prior myocardial infarction and prior chronic kidney disease), and the Killip Kimball functional class at admission. Other variables: reperfusion therapy (fibrinolysis or PCI), hospital stay, in-hospital complications and discharge state (alive or dead), as well as several laboratory exams (creatinine, triglycerides, FPG and WBC) were registered. The investigation was in accordance with the ethical principles set out in the Declaration of Helsinki and was approved by the Ethical Committee in Enrique Cabrera Teaching General Hospital.

### Definition of LGI and final endpoint

The construct LGI was calculated based on fasting WBC and FBG values obtained at admission as previously reported: FGB (mmol/L) * 18 * WBC (10^9^/L)^14-20^. In-hospital complications were the final endpoint: mechanics, arrhythmias, pericarditis, heart failure, cardiogenic shock, angina, reinfarction, among others, except death.

### Statistical analysis

The information was processed by the statistical packet IBM SPSS version 19.0.0. Continuous variables were presented as mean ± standard deviation and differences between the two groups were examined by independent-sample t-test. Categorical variables were described as counts (percentages) and compared by Pearson chi-square test (Pearson χ^2^ test) or Fisher’s exact test appropriately. A *p* value<0.05 with the 95% confidence interval was regarded as statistically significant. The performance of LGI was determined by the discrimination and the calibration: the discrimination was evaluated by the statistical *C*, also known as area under curve ROC; the method utilized for calibration was the Hosmer–Lemeshow test. The Youden index was utilized to determine the optimal cutoff point value of LGI for predicting primary endpoint. An analysis of logistic multivariate regression was accomplished to determine if the ILG was an independent predictor of complications, as well as identify others. Initial variables with good correlation in univariate analysis with the final outcome, were included in the multivariate model. After selecting the model of logistic binary regression, a predictive scale was created according to coefficients of every predictor. The new predictive model created in the universe of study was tried in a population of validation.

**Table 1:** Baseline clinical characteristics of patients with and without in-hospital complications Data are presented as the mean (SD) or n (%). Italic values indicate statistically significant associations. Abbreviations: CKD, chronic kidney disease; FPG, fasting plasma glucose; KK, Killip-Kimball classification; MI, myocardial infarction; LGI leukoglycemic index; PCI, percutaneous coronary intervention; SD, standard deviation; TG, triglycerides; WBC, white blood count.

Variable	Total (n = 900)	In-hospital complications: Present (n = 450)	In-hospital complications: Absent (n = 450)	*p*
LGI	1471.5 (1066.2)	1651 (1236.6)	1291.9 (825.9)	*< 0.001*
Age, years	66.9 (11.7)	69.0 (11.2)	64.7 (11.8)	*< 0.001*
Sex, male	615 (68.3)	304 (67.6)	311 (69.1)	0.616
Medical history				
Hypertension	710 (78.9)	374 (83.1)	336 (74.7)	*0.002*
Diabetes	218 (25.6)	127 (28.2)	91 (20.2)	*0.005*
Smoking	462 (51.3)	207 (46.0)	255 (56.7)	*0.001*
Dyslipidemia	50 (5.6)	23 (5.1)	27 (6.0)	0.561
Prior MI	63 (7.0)	39 (8.7)	24 (5.3)	0.050
Prior CKD	25 (2.8)	18 (4.0)	7 (1.6)	*0.040*
Clinical presentation and Laboratory results				
KK ≥ II	236 (26.2)	224 (49.8)	12 (2.7)	*< 0.001*
Creatinine, µmol/l	102.5 (53.7)	111.5 (62.1)	93.5 (42.1)	*< 0.001*
TG, mmol/l	1.4 (0.9)	1.4 (0.9)	1.4 (0.9)	0.300
FPG, mmol/l	7.5 (4.1)	8.0 (4.3)	7.0 (3.1)	*< 0.001*
WBC, 109/l	10.8 (4.6)	11.4 (5.8)	10.2 (2.8)	*< 0.001*
Reperfusion therapy and Discharge				
Fibrinolysis	521 (57.9)	255 (56.7)	266 (59.1)	0.458
PCI	22 (2.4)	14 (3.1)	8 (1.8)	0.195
Death	71 (7.9)	69 (15.3)	2 (0.4)	*< 0.001*
Hospital stays, days	7.5 (3.6)	7.9 (3.9)	7.1 (3.1)	*0,002*

## RESULT**S**

### Baseline characteristic of study population

A total of 900 patients were included in the universe of study, with a mean (SD) age of 64 (12) years. Clinical characteristics and laboratory results of the total population and groups stratified by the occurrence of primary endpoint event were presented in Table 1. LGI was significantly higher in patients with primary endpoint compared those without. The complicated patients showed higher age, higher prevalence of hypertension, diabetes mellitus, CKD, and a Killip-Kimbal functional classification ≥ II at admission, but lower prevalence of smoking. In terms of laboratory indicators, participants with the endpoint event had higher levels of creatinine, FPG and WBC. It was observed significant differences among both groups of patients when taking into account mortality and hospital stay. ROC curved analysis of LGI as predictor of in-hospital complications showed a poor calibration according to Hosmer-Lemeshow test (χ^2^=21.208; *p*=0.006); its discriminating capability also was poor, with an area under curved (AUC) of 0.623 (95% CI 0.586–0.659; *p*<0.001). The LGI of 1188 was calculated as the optimal cutoff point for predicting primary outcome by Youden index, with a sensitivity of 60% and a specificity of 61.6%. Baseline characteristics of groups according to the optimal cutoff point of LGI were summarized in Table 2. Compared with patients in lower LGI group, those with higher LGI seemed to be women, higher prevalence of diabetes and presented a Killip-Kimball classification ≥ II. All of the laboratory values were significantly higher in patients with higher LGI. Finally, a significant incidence of the primary event in the group with elevated LGI was observed; a higher value was significantly associated with a higher risk of to develop an in-hospital complication [RR (IC 95%) = 2.4 (1.804–3.080); *p<0.001*].

**Table 2:** Baseline clinical characteristics of patients stratified by the optimal cutoff point of leukoglycemic index Data are presented as the mean (SD) or n (%). The groups were stratified by the optimal cutoff point of LGI determined by ROC curve analysis. Italic values indicate statistically significant associations. Abbreviations: CKD, chronic kidney disease; FPG, fasting plasma glucose; KK, Killip-Kimball classification; MI, myocardial infarction; LGI leukoglycemic index; PCI, percutaneous coronary intervention; SD, standard deviation; TG, triglycerides; WBC, white blood count.

Variable	LGI < 1 188 (n = 455)	LGI ≥ 1 188 (n = 445)	p
Age, years	66.4 (11.6)	67.4 (11.8)	0.208
Sex, male	325 (71.4)	290 (65.2)	*0.044*
Medical history			
Hypertension	347 (76.3)	363 (81.6)	0.051
Diabetes	64 (14.1)	154 (34.6)	*< 0.001*
Smoking	247 (54.3)	215 (48.3)	0.073
Dyslipidemia	19 (4.2)	31 (7.0)	0.068
Prior MI	32 (7.0)	31 (7.0)	0.969
Prior CKD	8 (1.8)	17 (3.8)	0.060
Clinical presentation and Laboratory results			
KK ≥ II	81 (17.8)	155 (34.8)	*< 0.001*
Creatinine, µmol/L	97.0 (59.1)	107.9 (47.1)	*0.002*
TG, mmol/L	1.3 (0.9)	1.5 (0.9)	*0.005*
FPG, mmol/L	5.6 (1.0)	9.5 (5.1)	*< 0,001*
WBC, 109/L	9.1 (1.8)	12.5 (5.8)	*< 0,001*
Reperfusion therapy and Discharge			
Fibrinolysis	255 (56.0)	266 (59.8)	0.257
PCI	12 (2.6)	10 (2.2)	0.705
In-hospital complication	180 (39.6)	270 (60.7)	*< 0,001*
Hospital stay, days	7.2 (3.2)	7.8 (3.9)	*0.027*
Death	15 (3.3)	56 (12.6)	*< 0,001*

**Table 3:** Univariate and multivariate analysis and predictors of in-hospital complications Italic values indicate statistically significant associations. Abbreviations: CKD, chronic kidney disease; FPG, fasting plasma glucose; KK, Killip-Kimball classification; MI, myocardial infarction; LGI leukoglycemic index; PCI, percutaneous coronary intervention; SD, standard deviation; TG, triglycerides; WBC, white blood count.

Variable	Univariate analysis: Exp β (95 % CI)	Univariate analysis: *p*	Multivariate analysis: OR (95 % CI)	Multivariate analysis: *p*
LGI	1.000 (1.000 – 1.001)	*< 0,001*	0.561 (0.407 – 0.773)	*< 0,001*
Age	1.034 (1.022 – 1.046)	*< 0,001*	1.024 (1.009 – 1.039)	*0.002*
Sex	1.075 (0.811 – 1.423)	0.616		
Hypertension	1.670 (1.206 – 2.312)	*0.002*	0.647 (0.432 – 0.967)	*0.034*
Diabetes	1.551 (1.140 – 2.111)	*0.005*		
Smoking	0.651 (0.501 – 0.847)	*0.001*		
Dyslipidemia	0.844 (0.476 – 1.495)	0.561		
Prior MI	1.684 (0.995 – 2.851)	0.052		
Prior CKD	2.637 (1.090 – 6.377)	*0.031*		
KK ≥ II	36.177 (19.804 – 66.086)	*< 0,001*	0.033 (0.018 – 0.60)	*< 0,001*
Creatinine	1.010 (1.006 – 1.014)	*< 0,001*		
Triglycerides	0.925 (0.799 – 1.072)	0.302		
Fibrinolysis	0.905 (0.694 – 1.179)	0.458		
PCI	1.774 (0.737 – 4.271)	0.201		

### Risk factors for in-hospital complications

**Figure 1:**
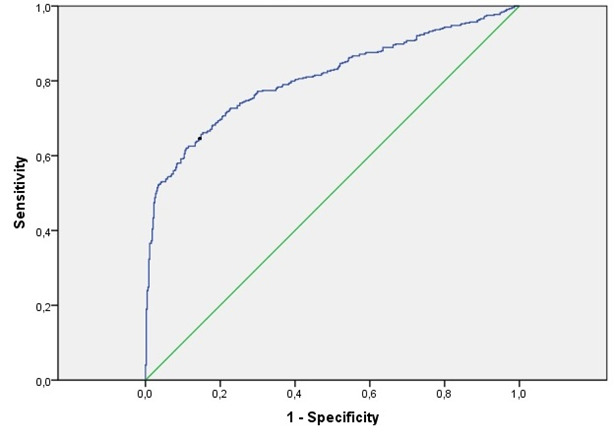
The receiver operating characteristic (ROC) curve of the predictive scale as a marker to predict in-hospital complications in STEMI patients

Univariate and multivariate analysis results and predictors for in-hospital complications are presented in Table 3. Univariate analysis revealed that LGI≥1188, age, hypertension, diabetes mellitus, smoking, Killip-Kimball ≥ II and creatinine were risk factors of in-hospital complications in patients with STEMI. After adjusting potentials confounding factors, the multivariate analysis found that the LGI≥1188, age, Killip-Kimball functional classification ≥ II and hypertension were independent predictors of primary event. This model had a good calibration (χ^2^=8.964; *p*=0.35) and its AUC for predicting the occurrence of complications was 0.808 (95% CI 0.778–0.837; *p*<0.001), which is considered very accurate (Figure 1).

### Development of a new predictive model of in-hospital complications

The coefficients of logistic regression obtained were transformed to create a new predictive scale, called “Predictive Scale of Infarction Complications”, abbreviated with the acronym PSIC (Table 4). This scale is integrated by four components: age≥66 years, LGI≥1188, Killip-Kimball classification ≥ II and a previous history of hypertension. The sum of the punctuation of this predictive scale in each patient varies from 0 to 6 points. With the new predictive scale, categories of risk were prepared according to the sum of these variables. Categories of risk were divided into lower risk (0–2 points), moderate risk (3–4 points) and higher risk (5–6 points) of in-hospital complications. The relation of these categories with the occurrence of complications in the universe of study is showed in the Table 5; it proved to be statistically significant (χ^2^=261.882; *p*<0.001).

### Validation of the model “Predictive Scale of Infarction Complications”

The lethality in the validation population was 1.3%, and 63 patients had in-hospital complication (27%). The performance of the PSIC new model had a good calibration (Hosmer Lemeshow test; χ^2^=12.67; *p*=0.12) and a good discrimination (AUC 0.70; 95% CI 0.619–0.768; *p*<0.001). Once more the validation population was divided according to the categories of risk predetermined for the PSIC model, and it was related with the occurrence of complications. This association proved to be again significant (χ^2^=28.19; p<0.001), the same result observed in the study population (Table 6).

## DISCUSSION

**Table 4:** Predictive scale of in-hospital complications in STEMI Abbreviations: STEMI, ST segment elevation myocardial infarction.

Variable	Punctuation
Age ≥ 66 years	1 point
Leukoglycemic Index ≥ 1 188	1 point
Killip-Kimball ≥ II	3 points
Hypertension	1 point

**Table 5:** Relation between categories of risk and in-hospital complications ^a^ Percent calculated of the patients’ total in each category of risk ^b ^Percent calculated of the total of the column

Category of risk	In-hospital complications: Present (n; %)a	In-hospital complications: Absent (n; %)a	Total (n; %)b
Low risk	153; 29.1	373; 70.9	526; 58.4
Moderate risk	113; 62.4	68; 37.6	181; 20.1
High risk	184; 95.3	9; 4.7	193; 21.4
Total	450; 50.0	450; 50.0	900; 100

**Table 6:** Relation between categories of risk and in-hospital complications in validation’spopulation ^a^ Percent calculated of the patients’ total in each category of risk ^b^ Percent calculated of the total of the column

Category of risk	In-hospital complications: Present (n; %)^a^	In-hospital complications: Absent (n; %)^a^	Total (n; %)^b^
Low risk	53; 23.8	170; 76.2	223; 95.7
Moderate risk	4; 100	0	4; 1.7
High risk	6; 100	0	6; 2.6
Total	63; 27.0	170; 73.0	233; 100

In the present investigation, values of LGI were statically higher in the patients with in-hospital complications compared with those without. However, when performance was calculated, its discriminative capability was hindered.Quiroga et al. were the first investigators who proposed the LGI as a scoreboard of fatal prognosis and in-hospital complications in patients with STEMI^10^. In the study, they correlated LGI with fatal development, heart failure and angina (*p*<0.001); the value obtained by AUC was 1600. There were several elements that do not permit comparison with our investigation: 1) they excluded the STEMI with admission higher than 48 hours from the onset of symptoms. The authors justified the methodology due to the complications that arise the first 48 hours after ischemia begins; 2) glycemia values utilized for the LGI calculation were obtained in the admission. In our study we utilized the FPG, into account studies which related the occurrence of complications with the glycemia of admission so much as FPG^4^; 3) they only included death, heart failure and post-infarction angina as complications; and finally, 4) the authors did not define in their study the AUC, sensitivity or specificity. The first study accomplished in Cuba about predictive capability of LGI was published by Lion-Aliz et al ^17^. The authors evaluated LGI as a prognosis marker in 128 patients that suffered STEMI and were admitted between January 2009 and October 2010 in Celestino Hernández Robau Hospital. Major adverse cardiovascular events (MACE) were defined as Killip-Kimball ≥ II, supraventricular and ventricular arrhythmias, conduction disturbance and reinfarction or postinfarction angina. AUC obtained was 0.682 (95% CI 0.590-0.775; *p*<0.001), with an optimal cutoff t of 1158; this area is considered a poor discrimination, same it was described in our investigation, although we obtained a lightly higher cutoff point. When the authors performed a logistic regression multivariate analysis, they demonstrated that age≥75 years, systolic blood pressure<100 mm Hg and LGI≥1158 were independent fatal predictors of in-hospital complications or death, or both.The present investigation aims to improve the prognosis of patients who suffer STEMI. The objective evaluation of the gravity of the disease through the punctuation system of the scale PSIC permits to identify patients with a high risk, who can benefit by invasive coronary therapeutic.

## **C**ONCLUSIONS

The LGI has a low predictive capacity for evaluating the risk of in-hospital complications of acute myocardial infarction. The new model “Predictive Scale of Infarction Complications” has a good performance which allows its application for the estimation of risk. The new model would be able to contribute the systems of health of developing countries of a clinical instrument without cost additional; it will permit predicting which ones the patients have bigger risk of an adverse outcome.
